# Primary Mediastinal Small Cell Neuroendocrine Carcinoma Presenting with Superior Vena Cava Syndrome

**DOI:** 10.7759/cureus.4873

**Published:** 2019-06-10

**Authors:** Lina R Costanzo, Tariq Kewan, Kevin Kerwin, Hamed Daw

**Affiliations:** 1 Internal Medicine, Ohio University College of Osteopathic Medicine, Warrensville Heights, USA; 2 Internal Medicine, Cleveland Clinic - Fairview Hospital, Cleveland, USA; 3 Internal Medicine, Cleveland Clinic, Cleveland, USA; 4 Hematology and Oncology, Cleveland Clinic - Fairview Hospital, Cleveland, USA

**Keywords:** superior vena cava syndrome, mediastinal mass, neuroendocrine tumor, small cell neuroendocrine carcinoma

## Abstract

A mediastinal small cell neuroendocrine carcinoma (MSCNC) is a rare form of neuroendocrine malignancy. Diagnosis is challenging and requires pathological identification and imaging studies. These tumors are aggressive and recurrence and metastases frequently complicate patient management. Here, we present a case of superior vena cava (SVC) syndrome secondary to circumferential compression by a primary MSCNC.

## Introduction

Primary mediastinal small cell neuroendocrine carcinoma (MSCNC) is a very rare form of neuroendocrine malignancy. With only a few cases currently documented in the literature, the etiology of MSCNC remains unknown. These types of tumors are frequently discovered secondary to their compression of neighboring mediastinal structures [1]. Diagnosis requires both pathological identification via biopsy revealing the characteristic organoid pattern as well as immunohistochemical positivity for characteristic markers of MSCNC, including chromogranin A, synaptophysin, and neuron-specific enolase, among others [2]. Historically, these tumors are difficult to treat and interventions that initially appear to be effective frequently result in tumor recurrence and metastases.

## Case presentation

**Figure 1:**
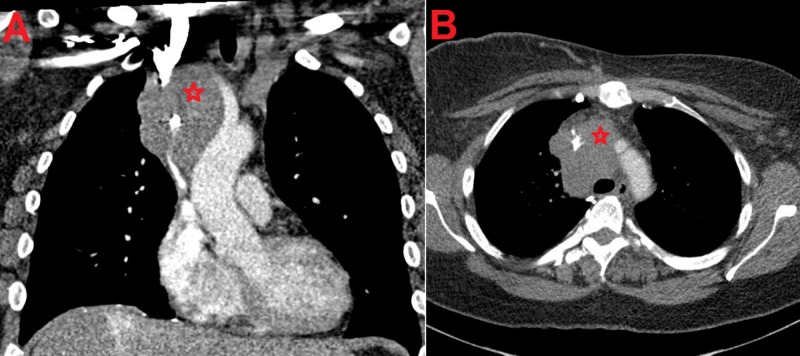
Computed tomography of the chest with intravenous contrast at presentation Coronal (A) and axial (B) section of the chest showing the circumferential compression of the superior vena cava by the anterior mediastinal mass (red star)

A 54-year-old female presented with a three-week history of facial swelling. The patient was treated on two occasions for suspected sinusitis, without improvement. The swelling continued to worsen and eventually progressed to affect her neck and bilateral upper limbs with associated fever, sore throat, dysphagia, shortness of breath, orthopnea, and 10-pound weight loss in the previous 10 months. She also reported new onset headache, lightheadedness, and blurred vision. On physical exam, the patient was noted to have congestive-appearing swelling of the face, neck, and bilateral upper extremities, along with dilation of superficial veins of the chest. Chest X-ray showed a new soft tissue density in the right paratracheal region and subsequent computerized tomography (CT) scan of the chest revealed a 6.2 x 4.9 x 8.2 cm infiltrative soft tissue mass in the right upper and middle mediastinum with circumferential encasement of the SVC and right subclavian artery and compression of the ascending aorta and aortic arch (Figure 1A-1B). Multiple left axillary lymph nodes with soft tissue stranding were also noted. Computed tomography (CT) scan did not show any lung lesions.

**Figure 2:**
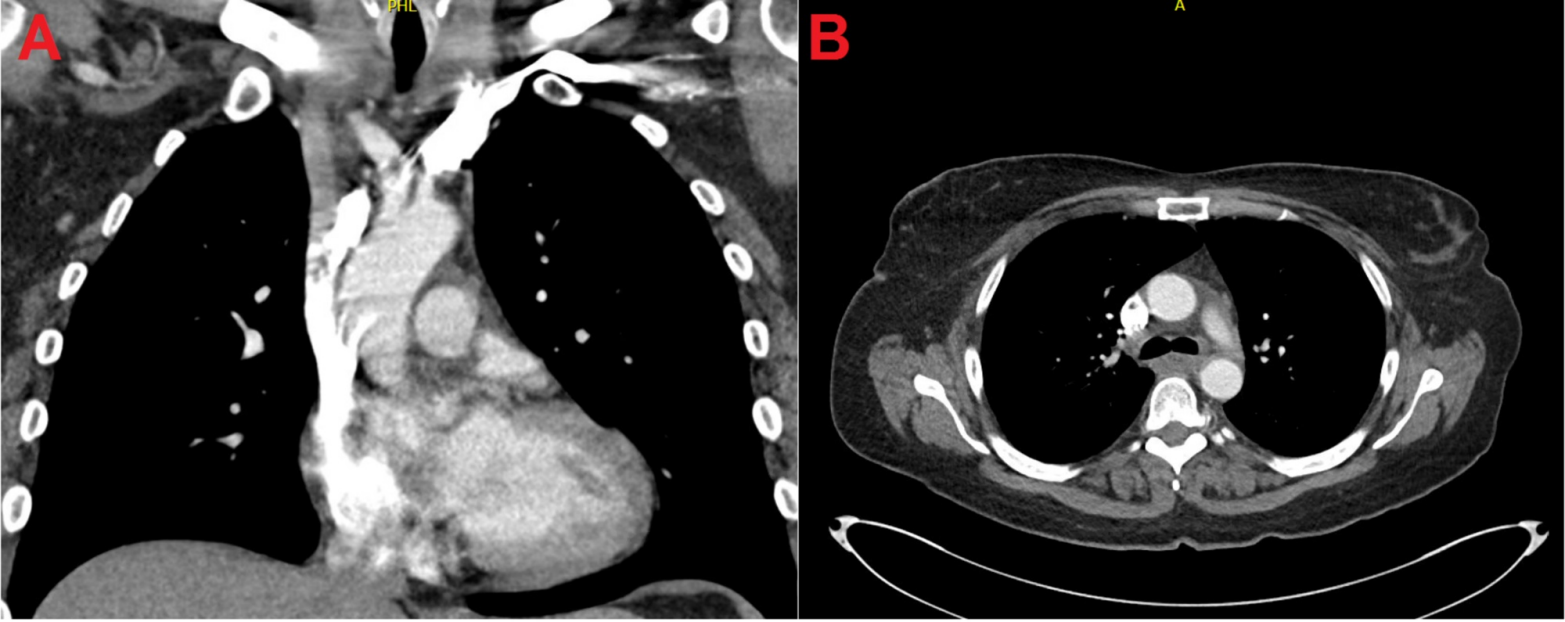
Computed tomography of the chest with intravenous contrast after treatment Coronal (A) and axial (B) section of the chest showing resolution of the mediastinal mass after chemoradiotherapy

Right paratracheal lymph node biopsy by mediastinoscopy identified the tumor as a primary small cell carcinoma. Immunohistochemical staining showed positivity for keratin AE1/AE3, synaptophysin, chromogranin, and thyroid transcription factor-1 (TTF-1). Subsequent magnetic resonance imaging (MRI) studies did not identify any areas of metastases. The patient received four cycles of cisplatin and etoposide chemotherapy with concurrent radiation therapy after the second cycle of chemotherapy. She also received whole brain prophylactic radiation. CT scan of the chest after chemoradiation showed a mild residual soft tissue mass in the right superior mediastinum and right paratracheal region compatible with a satisfactory response to chemoradiation therapy (Figure 2A-2B). The patient is still alive after six months of follow-up.

## Discussion

Neuroendocrine carcinoma can arise from many organs. The most commonly reported tumors arise from the lungs, thymus, parathyroid glands, ovaries, and gastrointestinal tract. A primary MSCNC is a very rare form of neuroendocrine malignancy [3-4]. Few cases are documented in the literature. A primary MSCNC is reported to be more common among men, with a mean age at onset of 54 years [5]. In our report, the patient was female and 54 years old.

A primary MSCNC may present like any other mediastinal mass. Patients may present with compressive symptoms or the tumor may be discovered incidentally by imaging studies done for other purposes. Malignant mediastinal tumors tend to invade nearby structures and patients may present with shortness of breath, hoarseness, stridor, or SVC syndrome [6]. SVC syndrome is caused by gradual compression of the SVC, leading to edema and retrograde blood flow [7]. Symptoms may include cough, dyspnea, dysphagia, and swelling or discoloration of the neck, face, and upper extremities. Often, collateral venous circulation causes the distension of the superficial veins in the chest wall [7-8]. In one case report, the patient presented with chest distress, dizziness, and a red face and was found to have SVC syndrome secondary to primary MSCNC [2]. Our patient presented with SVC syndrome associated with facial swelling, facial redness, upper extremity swelling, dysphagia, and shortness of breath.

The diagnosis of MSCNC requires both pathological identification via biopsy revealing the characteristic organoid pattern of the tumor, as well as immunohistochemical positivity for the characteristic markers of MSCNC [2,9-11]. Chromogranin A and synaptophysin are currently considered the most specific immunohistochemical markers for neuroendocrine tumors. The basal panels of immunohistochemical markers, such as caudal type homeobox (CDX-2), ISL LIM homeobox 1 (ISL-1), TTF-1, and paired box 6/8 (PAX 6/8) are currently being used to identify neuroendocrine neoplasms [2,10]. A large tumor located in the anterior or middle mediastinum showing scattered punctate calcifications and compressing the adjacent mediastinal structures in the CT scan of the chest should raise suspicion for a small cell neuroendocrine carcinoma [11]. Our patient had a right paratracheal lymph node biopsy by mediastinoscopy that showed a primary small cell carcinoma positive for keratin AE1/AE3, synaptophysin, chromogranin, and TTF-1.

No clear guidelines for MSCN treatment have been established due to the rarity of the disease. Surgical treatment complemented with chemotherapy and radiotherapy might alleviate the MPSCN patients’ symptoms and prolong their survival [2]. The prognosis of patients with primary neuroendocrine tumors remains poor due to the frequency of local recurrence and metastasis after surgical excision. Small cell carcinomas have the worst prognosis of primary mediastinal tumors and the shortest median survival time of approximately 14 months [12]. Our patient initially received two cycles of cisplatin and etoposide chemotherapy and later received another two cycles of chemotherapy with concurrent radiation. 

## Conclusions

A primary MSCNC is an extremely rare tumor. The disease can present with SVC syndrome or compression of nearby structures. Platin-based chemotherapy with concurrent radiotherapy improves patient's symptoms and may prolong survival.
